# Neuroarchitecture Assessment: An Overview and Bibliometric Analysis

**DOI:** 10.3390/ejihpe11040099

**Published:** 2021-11-05

**Authors:** Hessam Ghamari, Nasrin Golshany, Parastou Naghibi Rad, Farzaneh Behzadi

**Affiliations:** 1 Interior Design Program, Department of Family and Consumer Sciences, College of Health and Human Development, California State University Northridge, Northridge, CA 91330, USA; 2 Department of Architecture, University of Oregon, Eugene, OR 97403, USA; nasring@uoregon.edu; 3 Institute of Medical Science and Technology, Shahid Beheshti University (SBU), Tehran 19839 69411, Iran; parastou.naghibi.rad@gmail.com; 4 School of Architecture and Environmental Design, Iran University of Science and Technology (IUST), Tehran 13114 16846, Iran; behzadii.farzane@gmail.com

**Keywords:** neuroscience, architecture, neuroarchitecture, bibliometric analysis, VOSviewer, environmental design

## Abstract

Research on the relationship between architecture and neuroscience has increased in number and significance since the 1990s. Although a growing number of studies revolve around this field of research, there are very limited studies that have reviewed and assessed the field and there is a gap in the literature to address the overall analysis of neuroarchitecture literature and its evolution. Additionally, neuroarchitecture literature is now challenging to manage because of its multidisciplinary scope and wide range spread within different themes and journals. The primary aim of this study is to present a bibliometric analysis of three decades of research on neuroarchitecture. This provides an overall picture of the field and its research landscape. Two hundred and ninety-five publications were included in the final database of the study after screening processes. Next, a science mapping tool, VOSviewer, was utilized to detect major topics as well as influential authors, countries, publications, and prominent journals using different network analysis techniques such as term co-citation, term co-occurrence, and bibliographic coupling. Next, a similar co-occurrence analysis was conducted to identify the major themes and the evolution of the intellectual basis of the field. SciMAT was also used to detect how the intellectual base of the knowledge in the field has evolved over time. It also assisted to identify the major themes that have contributed to this evolution. The results show that this field has initially been mainly focused on few themes but has later become more diversified to acknowledge the multi-faceted characteristics of neuroarchitecture; over time, the intellectual base of the field of neuroarchitecture started to grow, particularly from 2016. Major progress in the development of theoretical and methodological approaches has been achieved and there has been a paradigm shift toward major keywords in neuroarchitecture such as EEG, fMRI, and virtual reality.

## 1. Introduction

In various environments, people are exposed to a vast number of architectural features that impact their well-being during their daily lives, and they respond cognitively as well as emotionally to built environments [1]. How people behave and how their mood changes in different spaces are directly linked to the architectural qualities of built environments [2]. These affect humans at the cognitive (understood as the processing and appraisal of perceived information) and also the emotional level (understood as the adaptive reactions to the perceived information), which both operate through closely interrelated systems [1,3].

Experts who are interested in environmental psychology have studied the influences of architectural structures on human behavior and emotion through empirical studies since the early 20th century. Therefore, individuals are becoming well-informed of the relationships between the built environment and their psycho-physiological well-being [4]. The literature related to environmental psychology has shown the importance of environmental design and also its impression on individuals’ quality of life. In other words, the way people perceive and experience built environments are considered the most prominent factor in the literature. Multiple studies in social sciences have explored the impact of built environments on human psychological health [5,6,7,8].

Accordingly, while architects and urban planners provide a context for various human experiences by designing diverse environments [4], a serious gap is observed at the intersection of architecture and neuroscience, which is called neuroarchitecture. There is a lack of objective evidence proving the impact of the built environments on the human brain. Thus, the field of neuroarchitecture concentrating on the significance of understanding subconscious neurophysiological reactions to environmental experiences emerged as a result of these studies in recent decades [9]. In this regard, recording human neural activities has been possible when they are exposed to architectural spaces by applying neuroscientific techniques [10,11]. Neuroscientific data play an important role in environmental behavioral studies and bridging the gap between architecture and psychology [12]. Current knowledge about basic mental processes such as visual perception, spatial navigation, and memory, which address our responses to architecture and the surrounding environment, supports this argument more than before [13]. Interestingly, neuroarchitecture uses neuroscientific tools to investigate the impact of different architectural styles on human perception and subjective experience [9]; therefore, recent progress in neuroscience has allowed scientists to improve and focus on the knowledge of human responses and has created a new way of thinking about current theories in the field of architecture and urban design as well [14].

This research concentrated on the studies carried out at the intersection of architecture, environmental psychology, and neuroscience. Surprisingly, these cross-disciplinary studies provide robust guidelines and frameworks for designers and architects to understand both the complex relationship between the built environment and humans and the impression of built environments on their emotions [2]. While there have been some improvements in this field, many researchers speculate that we are at the beginning of the evolution of cognitive research, neuroscience, and architecture [15]. Furthermore, the rapid growth of functional brain imaging methodologies has allowed cognitive neuroscience to address open questions in various fields of science [7]; as a result, a surge of research on early brain responses to architecture and urban design has been observed in recent years. Neuroimaging technologies, such as magnetic resonance imaging (MRI), functional magnetic resonance imaging (fMRI), electroencephalography (EEG), magnetoencephalography (MEG), and positron emission tomography (PET) provide increasing insight into how the human brain responds to architectural design features and how early experiences affect that response. Measuring and exploring brain activities are objective methods to assess the physiological and psychological impacts of engaging with environments [16]. Concerning brain mapping, the results of its recordings could indicate the user’s state of mind, which is a measurement of their mood, and it can indicate the level of engagement, interest, creative inspiration, attention, cognition, and focus [8,17,18,19]. These techniques also help scientists to identify neural networks involved in cognitive processes and each technique provides different information, which in turn illustrates merits and demerits of cost, safety, and the temporal and spatial resolution of using each of the techniques [20].

In recent years, several studies related to neuroarchitecture have been conducted from different architectural viewpoints, namely architectural styles [21], features of interior design [9,22,23], embodiment [24], contours [12], height, and enclosure [12], built vs. natural environments [25,26], lighting [27], color [28], façade design, and sunlight pattern [29,30], and also impacts of the built environment on human memory were investigated [31]. A few studies explored the relationship between natural landscapes and both psychological and physiological perspectives [32,33,34]. Tang et al. [32] illustrated a significant difference between restorative values of urban and natural environments. Applying natural features including water and mountains showed the most value in a comparison between the forest and urban landscapes: Aspinall et al. [33] showed there was the less frustration in green space zones and the higher levels of meditation while moving through them; in contrast, more engagement was observed while moving out of them. Neale et al. [34] suggested that there were significant neural activities in the brains of senior citizens experiencing different urban spaces and also these may represent the relation between subjects’ age and the condition of different urban spaces. The urban green space showed a restorative effect on older adults as well.

Chamilothori et al. [30] revealed that façade and sunlight geometrical patterns significantly influenced subjective responses. Participants showed a larger decrease in their heart rates while they were being exposed to irregular geometrical patterns compared to blinds. Naghibi Rad et al. [35] showed how different geometrical window shapes affect subjects’ neural activities, which were linked to whether they were pleasant or not by being exposed to the stimuli. Additionally, Higuera-Trujillo et al. [1] believed that neuroarchitecture provides a promising framework for future design and studies of the built environment and, also, they addressed the potential of neuroarchitecture and its precursors’ approaches in their scoping review.

A recent systematic review discussed how architectural environments emotionally and perceptually affect the human brain and also it emphasized the most current experimental procedures and brain imaging techniques applied in this field [35]. In this study, they have categorized articles into three subfields according to their task-space including: interior design, urban design, and building design. The results showed that fMRI and EEG have been used more than the other techniques in recent years, especially in neuroarchitectural studies revolving around human emotion, feeling, and perception.

In another systematic review [36], different environments affect brain activities and emotional responses were investigated. The findings showed low-frequency brain waves and also slight brain activities in the frontal lobe are related to natural environments while exposure to urban environments causes activation in the posterior cingulate cortex. This study also indicates that the validity of the subject’s responses improves when more senses are involved. While Norwood et al. [36] argued that there is a lack of sufficient studies investigating the impact of chronic exposure to environmental attributes on human brain activity and behavior, de Paiva and Jedon [37] in their review paper analyzed the results of research in the field of neuroarchitecture to find out how architecture influences individuals’ moods and behaviors by focusing on the short and long-term effects. This study revealed that in addition to physical features of the environment, “time/frequency of occupation and duration of the effect” are also influential attributes that need to be considered in microarchitectural studies.

While the number of experimental studies on neuroarchitecture as a multifaceted discipline has increased immensely, still lacking is a holistic review of the current state of neuroarchitecture literature and its evolution that addresses emerging concepts in the field. Several systematic reviews have been conducted to provide a framework for the existing knowledge in the field; however, there have been limited efforts to identify the current trends, map the evolution, and overview the research landscape in neuroarchitecture. The main objective of this study is to present a bibliometric analysis of about three decades of research on neuroarchitecture to provide a better understanding of knowledge, structure, and patterns between different items related to the field. Accordingly, this analysis provides a wide variety of information about the thematic analysis, and science mapping that helps to identify the most influential authors, universities, journals, and countries that have researched in the field. Interested readers are referred to [38] for more details regarding the bibliometric analysis. Results of such analyses can be used for different purposes. For instance, either they could be used to identify understudied topics and themes and analyze the impact of scientific literature, or researchers in the field can be referred to the outputs as a one-stop source to obtain a relatively quick understanding of the field literature and its structure. Moreover, it may be used to understand key areas that need to be more explored in the coming years, which in turn would help them in their research design process. It must be noted that bibliometric analysis is different from systematic review and it aims to show the overall landscape of the research and highlight major topics that have been dealt with generally and in different periods. Some of the sub-topics highlighted in this study warrant more detailed analysis that can only be achieved through a systematic review.

This paper is divided into four sections. After the introduction, the materials and methods are discussed in Section 2, in which methodological choices are applied in bibliometric analysis, and also parameters used to search are explained. The results of the bibliometric analysis are reported and also interpreted in Section 3. Section 4 presents the conclusions of the study by discussing the main emerging topics in the literature and evaluating key debates and research gaps in the field, limitations of the study, and recommendations for future studies.

## 2. Materials and Methods

The major phases of the study included creating the database and VOSviewer analysis. These phases are discussed here.

### 2.1. Creating Database

The first phase was to create a database that included the relevant studies of neuroarchitecture. For this purpose, a wide range of literature search strings was used that consisted of keywords relevant to neuroscience and physical environments. The search terms were selected based on the authors’ knowledge of the field and also the analysis of existing review papers on the topic. While designing the search string, it was also considered that different variants of terms may have been used in the literature.

TS = ((“architecture” OR “built environment” OR “architectural space” OR “interior space” OR “environment design” OR “physical environment”) AND (“neuroscience” OR “neuro architecture” OR “neuroimaging” OR “brain imaging” OR “brain responses” OR “neural activation” OR “neural responses”)).

The search results were limited to articles in addition to limiting to the topic section (title, keywords, and abstracts) to identify publications mainly focusing on neuroarchitecture. While this search might not have retrieved all related publications, it found the publications where researcher(s) had a main focus on neuroscience, architecture, and physical built environment for their publications. The search was conducted on 25 April 2021, based on the abstracts, keywords, and titles of research papers (only in English) indexed from 1965. There were 2565 papers retrieved from the following Web of Science (WoS) databases: Science Citation Index Expanded, Social Sciences Citation Index, Arts, and Humanities Citation Index, and Emerging Sources Citation Index. These databases were selected for two main reasons: (1) they are well-recognized in academia for archiving quality peer-reviewed research; (2) the software tools available for text mining and bibliometric analysis can only process bibliographic outputs generated by these databases.

Initial search findings returned some publications related to electrical engineering, robotics, and computer science systems, as terminology is shared between these research fields. Therefore, irrelevant publications that lacked a relationship with neuroarchitecture were filtered out from the database. Overall, 2408 publications were removed from the database after careful review of abstracts, keywords, and titles by the authors. Using the same keywords of the search string, a hand search was also conducted of different journals related to the field, including *Journal of Environment and Behavior*; *Health Environment Research and Design Journal*; *Journal of Environmental Psychology*; *Environment and Behavior*; *Journal of Experimental Psychology: Learning, Memory, and Cognition*; *Cognitive Psychology Journal*; and *Environment and Planning B: Urban Analytics and City Science*. Next, additional articles were further identified from cross-referencing of relevant articles. An additional 138 articles obtained via the hand search were added to the database.

**Figure 1:**
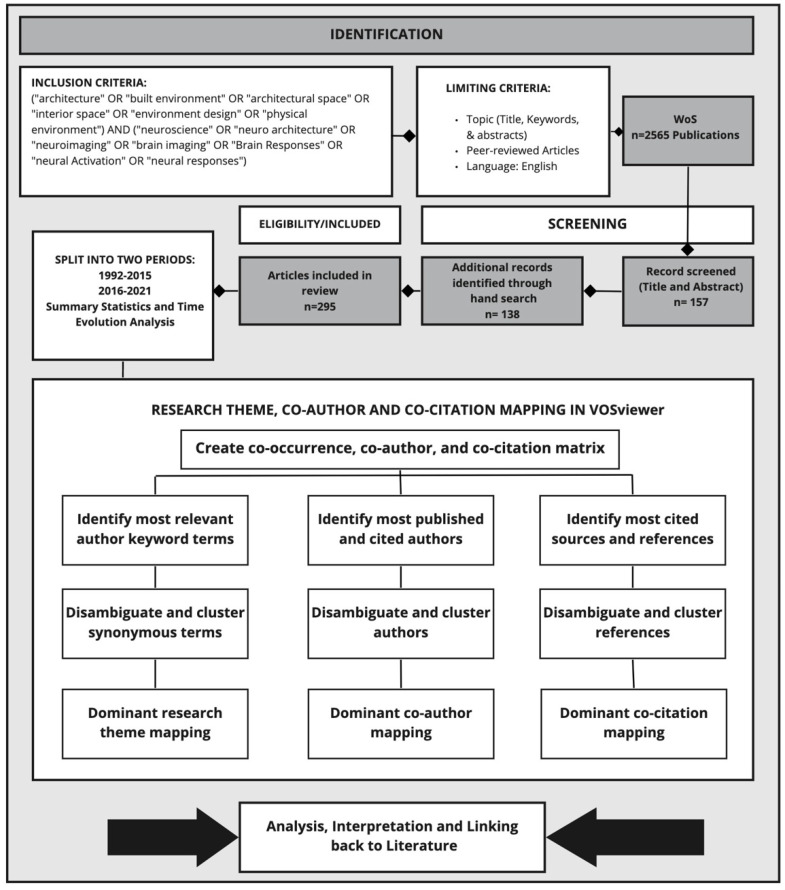
Search strategy and multifaceted bibliometric analysis framework (adapted from [39]).

The final database contained 295 publications (Figure 1). The database was reviewed to identify the temporal patterns in the literature including dividing it into two broad periods (initial research 1992–2015, and developing research 2016–2021). The periods were identified based on the distribution of publications to make sure there were sufficient data in each period for further analyses and comparisons. It also appears that there was a rise in the number of publications from 2015.

### 2.2. Analysis Using VOSviewers

Over the years, different visualization tools have been used for science mapping and bibliometric analysis [38]. The main objectives of these visualization tools are to demonstrate the dynamic and complex relationships between the fields, authors, journals, organizations, countries, and the core concepts of the knowledge. This study used VOSviewer, a widely-used tool that is simple and convenient to interpret in a graphic interface to create bibliometric networks of authors, publications, journals, organizations, and countries [40]. The networks are generated based on co-occurrence terms, co-authorship, bibliographic coupling, and co-citation analyses (readers interested in more details on these methods are referred to [41] for more details). To add more accuracy for the analyses, a thesaurus file was created and adopted in this study (e.g., gender differences and sex differences all referred to the same terminologies and were considered as sex differences).

In addition to the co-occurrence analysis, the bibliographic coupling by countries analysis was conducted to detect the leading countries that have worked in this research field. The “co-citation” analysis was also utilized to identify the most prominent journals and publications and demonstrate their relationships. The output of each of these analyses is a graph network of nodes and links, where larger nodes and thicker links indicate higher importance of those components. The maps generated by VOSviewer are based on the modularity-based clustering method that includes nodes and links. The node sizes demonstrate the frequency of the considerations, and the thickness of the links displays the strength of the connections between the nodes. Additionally, closely linked nodes form clusters that, for instance, in the case of term co-occurrence analysis indicate thematic clusters.

### 2.3. Analysis Using SciMAT

SciMAT is a freely available tool that can be used to detect the thematic and conceptual evolution of neuroarchitecture over the past three decades. SciMAT allows science mapping within a longitudinal time frame. In this study this tool is mainly used to detect the similarities and relationships between the terms by using the co-occurrence analysis. The results from SciMAT analysis were also compared with the bibliometric analysis from VOSviewer. The information regarding the modules and algorithms of SciMAT is available in [42]. Prior to conduct the analyses, the ‘word group manual set’ option was used to merge the synonymous and/or misspelled terms of the study. SciMAT uses the article keywords and keywords added by the indexing database for keyword co-occurrence analysis.

**Figure 2:**
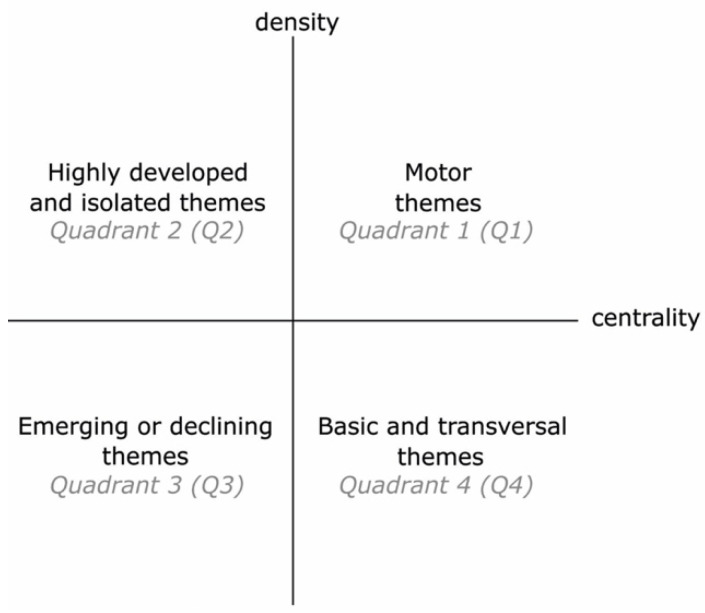
Strategic diagrams of SciMAT analysis (adapted from [38]).

According to the recommendation of [38], the ‘equivalence index’ was used to normalize keyword co-occurrence frequencies. For the clustering algorithm, the ‘simple centers algorithm’ was used. In order to quantify the importance of difference keyword clusters, h-index, average citations, and total citations were used. The main output from SciMAT analysis used for this analysis was strategic diagrams (Figure 2). A strategic diagram is a 2D plane in which the horizontal axis corresponds to network centrality and the vertical axis corresponds to density [38]. Centrality demonstrates the degree of interaction between networks. A higher centrality shows a stronger connection to other themes and possibly a more essential role in the evolution of the discipline. On the other hand, density is a measure that shows the degree of interaction within a network. A higher value of density for a theme shows that it has stronger internal ties and it is more developed [38]. According to the position of the plane, four types of themes can be identified [38], p. 150:

1. “Themes in the upper-right quadrant are both well developed and important for the structuring of a research field. They are known as the motor-themes of the specialty, given that they present strong centrality and high density. The placement of themes in this quadrant implies that they are related externally to concepts applicable to other themes that are conceptually closely related”.
2. “Themes in the upper-left quadrant have well developed internal ties but unimportant external ties and so are of only marginal importance for the field. These themes are very specialized and peripheral in character”.
3. “Themes in the lower-left quadrant are both weakly developed and marginal. The themes of this quadrant have low density and low centrality, mainly representing either emerging or disappearing themes”.
4. “Themes in the lower-right quadrant are important for a research field but are not developed. So, this quadrant groups transversal and general, basic themes”.

It should be noted that the interpretation of the themes in the lower-left quadrant (emerging or declining themes) requires a deep understanding of the research landscape of the field. It should also be explained that the size of nodes in the network is proportional to the frequency of documents associated with the keyword.

## 3. Results

The results of the term co-occurrence analysis and bibliographic coupling are presented in this section.

### 3.1. Thematic Clusters: Term Co-Occurrence Analysis

**Figure 3:**
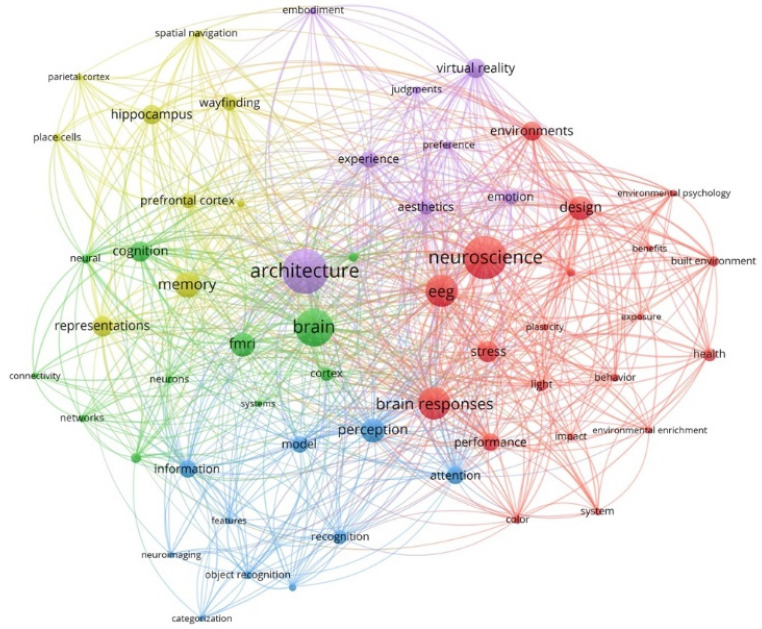
The output of the term co-occurrence analysis for the whole study period from 1992 to 2021 (node size is proportional to the number of terms).

Term co-occurrence is a bibliographic analysis method that creates thematic clusters by detecting the major focus areas and identifying topics/sub-topics that co-occur more frequently [41]. The node size is proportional depending on the number of times that the term has been used. Proximate nodes are more closely linked to each other and the link thickness connecting them is relevant to the strength of the connection [40]. The total link strength indicates the number of publications in which two keywords occur together as well. It should be noted that the interpretation of the term co-occurrences and thematic clusters should be presented cautiously since the frequency of the term co-occurrence and connection between the terms might not be sufficient to specify the relationships between the terms; therefore, knowledge of the related field is required. The results of the co-occurrence analysis generated by VOSviewer for a minimum threshold of 67 keywords are shown in Figure 3.

This section explains and analyses bibliometric results in the literature of neuroarchitecture and identifies the evolution of the core research topics over the years. Identifying thematic clusters was carried out by concentrating on strength of the connections between the terms in software. Besides, the minimum number of co-occurrences of each keyword was five for this analysis. The frequency of co-occurrence of the top 20 highest occurring terms is presented in Table 1.

Over 30 years from 1992 to 2021, terms including ‘architecture’ (*n* = 43), ‘neuroscience’ (*n* = 41), ‘brain’ (*n* = 36), ‘brain response’ (*n* = 30), ‘EEG’ (*n* = 30), and ‘memory’ (*n* = 24) had higher values of occurrence and total link strength. This shows that these terms have received more attention and are linked with other terms. Thus, it can be seen that these keywords are located near the borders of the clusters, which in turn indicate cross-cutting terms that have strong connections to different clusters and terms. Furthermore, higher values for terms such as ‘design’ and ‘brain response’ imply that a lot of attention has been paid to the studies conducted at the intersection of architecture and neuroscience. Although one of the main reasons for higher values in co-occurrence analysis for ‘architecture’ and ‘neuroscience’ terms is their inclusion in the search string, these terms are kept in the analysis because excluding them may result in the omission of important terms that are linked to these keywords. Higher values of the terms ‘fMRI’ and ‘EEG’ indicate the significant role of these neuroimaging techniques in neuroscience studies. It has also been proven that these neuroimaging techniques are the most common ones in the field of neuroarchitecture [35].

Figure 3 shows that five predominant clusters are identified based on the co-term occurrence analysis. The largest cluster (red) includes 20 keywords, which is centered on the concept of neuroscience. This cluster mainly revolves around the impact of architectural stimuli such as ‘color’ and ‘light’ on ‘brain responses and ‘health’ outcomes of humans. This cluster also shows a close relationship between ‘EEG’, ‘brain response’, and ‘design’ that shows the significant role of EEG in the literature of neuroarchitecture. There is also a connection between ‘health’, ‘stress’, and ‘environment’ terms. Several studies in neuroscience identified that access and exposure to natural environments are associated with improved health outcomes [36]. People experience less stress [43] and better general health [44] in a room with a natural environment compared to synthetic ‘nature’ [43].

The second cluster (green) that has mainly focused on brain structure and its function includes 11 keywords such as ‘brain’, ‘cortex’, ‘neuron’, ‘cognition’, and ‘fMRI’. ‘fMRI’ and ‘brain’ are often the main focuses of research because of their pronounced relationship to neuroarchitecture; also, fMRI is used to measure changes in neuronal activities in different areas of the brain by detecting changes during functioning and to create detailed photograph-like representations [14,45]. Several studies investigated the impact of the architectural environments on users’ state of mind and identified influences on different regions of the brain [46,47,48]. In a review carried out by [8] the impact of the built environment on the users’ state of mind was investigated with a focus on the measured brain activities to indicate the momentary state of mind.

The third cluster (blue) includes 10 keywords, and it is mainly related to ‘perception’, ’recognition’, ‘attention’, and ‘information’. It shows a close relationship between perception, attention, and model. In the literature, Lynch [49] conducted an empirical study of human experience associated with perception, cognition, and memory in the study of a city. According to [50], neuroscience is a section of life sciences studying the brain, nervous system, and brain processes, which includes sensation, perception, learning, memory, and movement.

The fourth cluster (yellow) consists of nine terms and mainly focuses on issues related to ‘memory’ and ‘representation’, which are often researched in conjunction with ‘hippocampus’ since they play an integral role in learning and memory. This cluster has strong links to the brain structure and regions, which indicate that they have an impact on our memory. Other key aspects of this cluster include ‘way finding’ and ‘spatial navigation too. Additionally, memory has played an essential role in indoor wayfinding literature, and different memory systems are responsible for the two types of spatial memories relevant to wayfinding [51]. Cubukcu [52] defined wayfinding as spatial knowledge about one’s current location, destination, and spatial relation between them. According to [51], the hippocampal system plays an important role in acquiring knowledge (cognitive map) and the caudate nucleus affiliated to route knowledge. The duty of the human central nervous system is collecting information from the entire body and coordinating activities across the whole organism to govern all forms of activities ranging from the heartbeat and involuntary reflexes to deep thoughts and creative ideas. Therefore, this coordination forms our perceptual memory, leading to our mental well-being [53,54,55].

The last cluster (purple) includes eight terms, and it is mainly related to architecture which strongly is linked with virtual reality (VR) in neuroscience studies. VR is an optimal tool to evaluate human responses to architecture at both behavioral and neurophysiological levels [1,56,57]. Proximity and strong linkages between these terms and the term of ‘experience’ show that special attention has been paid to the influences of built environments on human experiences, including behavioral, emotional, sensational, and cognitive responses covering conscious, unconscious, and subconscious experience [58]. The more we understand and identify human responses to built environments, the better we can design based on their priorities [14]. When it comes to human experiences in architectural spaces, keywords such as esthetics, emotion, preference, and judgment influence them. Neuroscientific and VR technology has been extensively used in experiments carried out in the fields of art and esthetics as well.

The related themes and the relationship between ‘architecture’ and ‘neuroscience’, which were the major keywords in the literature, were investigated by these analyses. Circles in the same color cluster suggested a similar topic among these publications; therefore, different colors referred to the clusters of themes that co-occurred frequently. While there were three major clusters, all of which were noteworthy, two of these were less dense. ‘Architecture’, ‘neuroscience’, and the brain structure term were mainly used in the context of the three dominant clusters colored red, green, and purple. Furthermore, in the co-occurrence map, red and purple clusters focusing on neuroarchitecture terms were well-connected, and also the green cluster was placed at the center of the visualization map of co-occurrence showing strong connections with other clusters. However, although the blue cluster was large, it was not well-connected with others.

### 3.2. Thematic Focus Transition over Time

One way to examine how the thematic focus of research has changed over the years is to identify keywords that frequently come up in the articles. Research hotspots in each period can be determined based on the frequency of keywords in articles and drilling down based on specific periods shows how some topics have fluctuated (upward, downward, and stable trends) over the years [39]. This part of the article discusses the results of the bibliometric analysis of neuroarchitecture literature and the evolution of the core research topics used over time. Although this research area is a novel field, it has progressively gained attention with an increasing number of studies conducted. Therefore, many efforts have been made to investigate various issues in the field from different aspects by publishing experimental and theoretical studies; of course, the number of reviews and theoretical publications is not as great as that of the experimental ones. This research area has been diversified with an increasing number of publications over time, and analysis shows that research carried out in the field of neuroarchitecture established a new area of scientific studies as well. The neuroarchitecture literature can be divided into two different periods: the preliminary period (1992–2015) and the developing period (2016–2021). Supporting quantitative details for these two periods are presented in Table 2.

**Figure 4:**
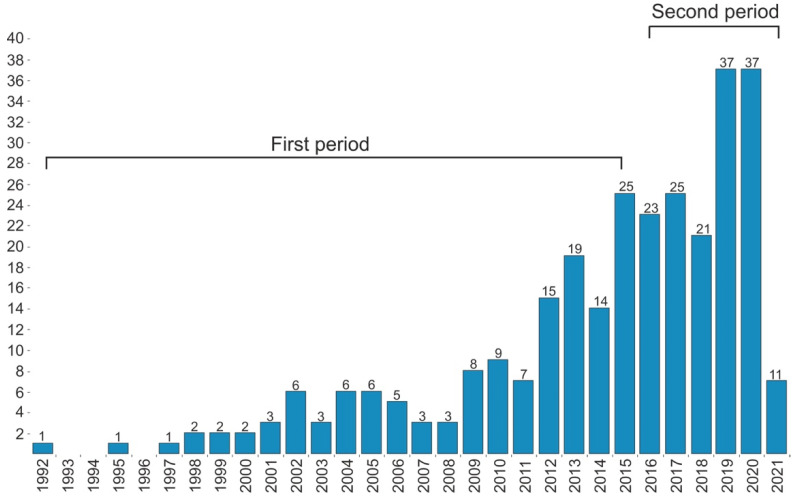
The number of articles published per year. Note that the lower number of publications in 2021 is because this study was conducted in early 2021. An upward trend is expected for 2021 and the following years.

The first published research mentioned in this mapping review dates back to 1992, which explored the impact of light (both natural and electric fluorescent light) on the production of stress hormones, student performance, body growth, and sick leave of school children. The findings of this literature mapping illustrate that there were 295 publications, of which 142 (47.8%) were carried out during the 23 years called the preliminary period (1992–2015), and 153 research studies (52.2%) were related to the developing period (2016–2021), during which rapid growth was observed in research in the field over a 5-year period. According to the results of literature mapping, there was a steady increase in the number of studies from 1992 to 2014, while the number of publications grew dramatically in 2015. This trend continued during the following years. Figure 4 demonstrates the rise of the number of publications in the field of neuroarchitecture during the past three decades.

**Figure 5:**
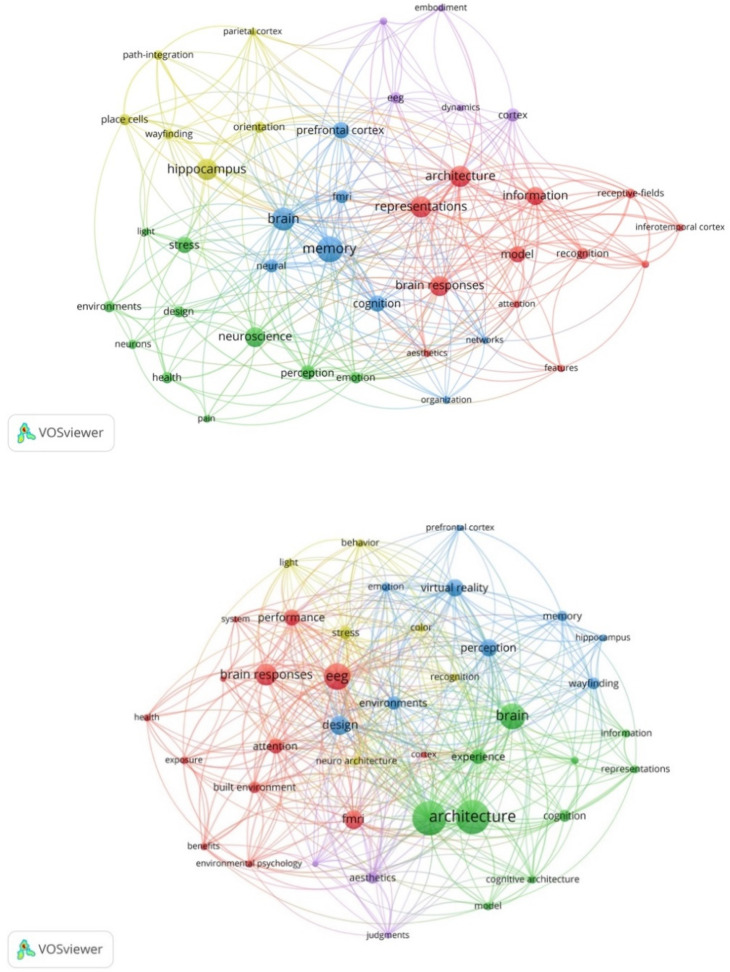
Major themes in the literature based on the term-occurrence for each of the two time periods: preliminary (1992–2015) and developing (2016–2021). The size of the circle is proportional to the occurrence of the keyword, while the line thickness is proportional to the strength of co-occurrence.

Figure 5 shows the output of the term co-occurrence analysis for the preliminary (1992–2015) and developing (2016–2021) periods. With regard to the analysis of the first period, different aspects of the brain were the main focus; in fact, the main idea of the publications in the first period focused on ‘brain’, ‘memory’, and ‘architecture’, while ‘neuroscience’ and ‘EEG’ have become the dominant subject areas through the second period. On the other hand, both ‘brain’ and ‘architecture’ had become key subject areas by the year 2021. The concentration of this research field has shifting from brain concepts to other concepts related to neuroarchitecture, such as ‘EEG’, ‘fMRI’, and ‘virtual reality’ from 2016 to 2021 within the second period. As for the developing period, the thematic focus of the field was remarkably diversified. In fact, this indicates enlargement of the intellectual base and may show a gradual paradigm shift toward recognizing neuroarchitecture as a multi-faceted concept with various aspects. The results demonstrate that studies in the preliminary period mainly focused on brain function in the process of human perception and cognition of the environment, while studies in the second period are more concentrated on topics related to architecture and design and their impact on the human brain’s function through neuroscience.

The number of shared keywords between these two distinct periods also grew rapidly, leading to interpretations of a certain level of consolidation of the keywords introduced in each period. This means that the evolution of the field still continues. ‘Brain’, ‘memory’, and ‘architecture’ are motor themes playing underlying roles in defining the structure of the field within the first period. ‘Brain’ provides a basis for defining ‘memory’, which is a fundamental term in this period; also, it can be clearly observed that ‘hippocampus’ and ’prefrontal cortex’ were brain themes that were well-developed; however, there were no strong external ties with the other themes.

Interestingly, in the top five research priorities through the periods, research concerning both ‘architecture’ and ‘brain’ has been the primary focus of attention in the literature. When it comes to the research focus during the periods, the topic ‘neuroscience’ went up from 7th rank (1992–2015) to first (2016–2021), ‘EEG’ moved from 20th rank (1992–2015) to 3rd (2016–2021), and ‘fMRI’ moved from 15th rank (1992–2015) to 7th (2016–2021). The idea of making a bridge between the human brain and architectural environments has been introduced in theoretical and methodological approaches, which have created a paradigmatic change [4]. Accordingly, neuroscience offers the opportunity to create new hypotheses in the field of design and architecture, which have not been explored owing to the lack of commonly accessible sophisticated tools, such as functional magnetic resonance imaging (fMRI) and electroencephalogram (EEG) for architects and designers [11]. ‘EEG’ and ‘fMRI’, as neuroscientific measurement (brain imaging) tools, have also emerged as core topics in the neuroarchitecture literature [35,59,60,61]. Munoz et al. [62] used a low-cost EEG to record and classify brain signals and emotions while architectural spaces were being displayed to subjects. Tiago-Costa et al. [63] investigated the relationship between thermal environments and the amplitude of brain waves (alpha and beta) by using EEG. In addition to EEG, fMRI has also been widely used in the second phase (developing period). A study conducted by Zhang et al. [47] compared human brain activities while color photographs of natural landscapes and landscape gardens were presented to subjects, using fMRI. They showed the contrast between appreciation of landscape gardens and natural landscapes was distinguished by stronger brain activities in the inferior occipital lobe, the left superior parietal lobe, the right fusiform gyrus, the right cuneus, and the right hippocampus. Other studies have highlighted the importance of using EEG and fMRI in neuroarchitecture studies [9,35,48,64,65]. According to [34], EEG was the most frequently as well as commonly employed neuroimaging technique according to the high temporal resolution of its signals, while fMRI studies were functional but not as common as EEG owing to restrictions in the implementation and laboratory settings, and the lack of portability in some tasks.

The topic ‘environments’ was ranked 24th in the first period (1992–2015), but it has become one of the topics that has been paid most attention as its rank shifted to 15th over the second period (2016–2021). This indicates how the interrelationships between neuroscience and environmental design have evolved over time. Frumkin et al. [66] detailed an agenda for future natural environments research and emphasized the importance of investigating the physiological mechanisms underpinning self-reported and observed psycho-emotional health outcomes. Furthermore, Norwood et al. [36] presented a systematic review to explore how different environments (man-made and natural) affect human brain activities and mood responses. Karakas et al. [14] also studied the influences of the built environment on human experiences based on the neuroscience approach by examining conceptualizations. In the preliminary period, there were some studies related to the keyword of ‘environments’ concentrating on mutual interaction between individuals and built environments [67,68]. They used traditional research techniques such as surveys and observations to obtain research pieces of evidence, while in the developing period, advances in science and technology such as the use of neuroscience and making a bridge between it and other sciences [4,69,70] assisted scientists to focus on new perspectives to expand the knowledge of the interactions between human and built environments.

‘Memory’, ‘brain’, ‘architecture’, ‘representation’, and ‘hippocampus’ were the most frequent and central keywords within the preliminary period (1992–2015), while ‘neuroscience’, ‘architecture’, ‘EEG’, ‘brain’, and ‘brain response’ have become the most frequent ones over the second period (2016–2021). Surprisingly, in comparison with the preliminary period, there has been far more emphasis on neuroarchitecture and neuroscientific tools between 2016 and 2021; however, the primary focus was mainly on brain characteristics including ‘memory’, ‘hippocampus’, and ‘representation’. The shift of this paradigm reflects better recognition of other characteristics such as ‘brain’, ‘brain response’, and ‘neuroscience’. Considering research and studies published during the developing period (2016–2021), the intellectual base has expanded and as a result terms with high centrality have emerged. Nancy Kanwisher and her associates in 1999 focused on the intersection between neuroscience and architecture in their studies [71]. They provided contexts to link the human brain and experiences being related to architecture and spaces [4]. Furthermore, the advancement of novel interdisciplinary research in the field of architecture and neuroscience was first introduced by John P. Eberhard, the founding director of ANFA (Academy of Neuroscience for Architecture), who took the first steps to define and develop the field of neuroarchitecture. The discussion of how neuroscience and architecture could join together was a subject opened up at the Salk Institute to discover the impression of built environments on the human brain and emotions. Accordingly, scientists could understand better how individuals experienced spaces [72] and how they were impressed by architectural features. Given these studies, it is obvious that the topic has widely received attention in the developing research phase (2016–2021). Psychophysical and perceptual explorations have established reciprocal links between architecture and brain responses. On the other hand, in the preliminary period, many studies focused on the brain. Arbib [73] introduced a neuromorphic architecture exploring ways to incorporate lessons ranging from studying biological brains to devise computational systems based on findings of neuroscience.

Concerning analyzing the results of the developing period (2016–2021), it appears that the intellectual base of the field expanded and consolidated around the major keywords mentioned in the preliminary period. Furthermore, high repetition and centrality of keywords related to brain-like response and EEG show that the research on neuroscience-based frameworks has been gaining more popularity, and has also become more comprehensive in recent years.

Research related to ‘esthetic’ shifted from 33rd rank to 16th from the preliminary (1992–2015) to the developing period (2016–2021). ‘Design’ (22nd to 6th) and ‘perception’ (13th to 9th) terms also remarkably increased in the literature since they were considered as a priority for researchers. On the other hand, downward trends are also identified for some topics, such as ‘memory’, which moved from the first rank to the 19th, ‘hippocampus’ (5th to 34th), ‘information’ (8th to 29th), and ‘representation’ (4th to 28th) between the two periods. The ‘frontal cortex’ (9th to 41st), ‘model’ (11th to 26th), and ‘cortex’ (16th to 39th) significantly lost their superiority in research studies; therefore, new preferable research topics have been established within the second period (2016–2021). Additionally, there are some other topics, such as ‘experience’ (11th), ‘built environment’ (15th), ‘neuroarchitecture’ (23rd), ‘cognitive architecture’ (25th), and ‘environmental psychology’ (30th), which were not in the first period but have appeared later in the developing research period. All these changes indicate that there is a huge shift from studying the brain function and structure in addressing emotional and behavioral outcomes (1992–2014) to investigating the impact of environment, architecture, and design on brain activity (2015–2021).

In the developing period, the topic of ‘virtual reality’ (8th rank) was a leading research priority. Virtual reality design has been used to rate the feeling of presence [24], investigate the effects of each sense on the presence, and compare the relaxing effects of viewing 3D versus 2D images of natural environments [74]. Researchers have used virtual reality as a new method to investigate the visual environmental factors that impact humans while experiencing architecture and space [9,12,30,75,76,77]. According to the results presented, not only the methodology but also the thematic base of the field has expanded. In fact, three decades of research resulted in improving the inclusion of different neuroarchitecture dimensions and aspects in the assessment process.

#### 3.2.1. Conceptual Structure and Evolution of the Field

SciMAT analysis results are presented as strategic diagrams that divide major themes of each period into four categories (motor themes, basic and transversal theme, highly developed and isolated themes, and emerging or declining themes). Descriptions of each category are presented in Section 2.3. The main results for each period are reported here.

##### First Period

**Figure 6:**
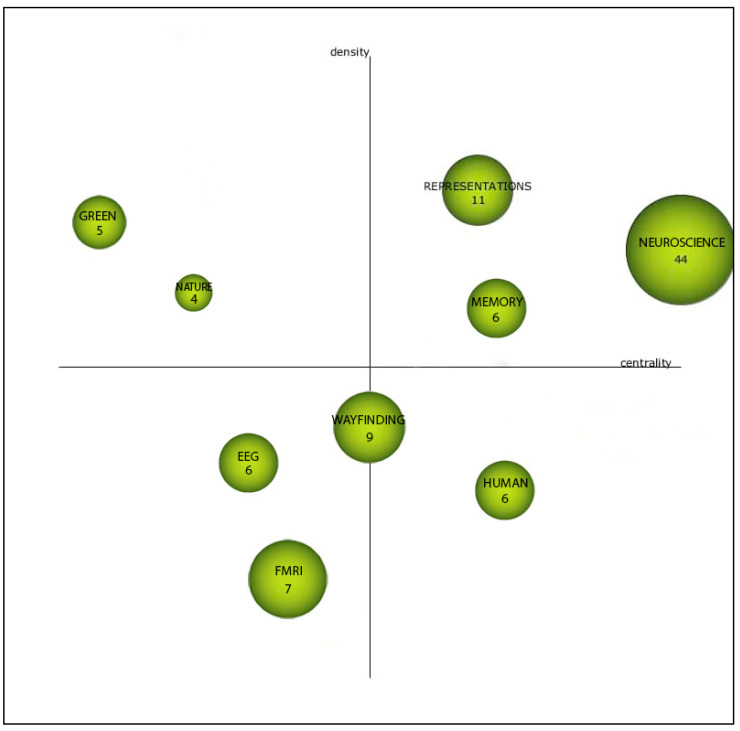
Major themes of the first period (strategic diagrams, see Section 2.3 and Figure 2 for description of the nodes and quadrants).

Figure 6 shows that during the first period (1992–2015) the intellectual base of the field was relatively limited and few major themes emerged from the SciMAT analysis. According to this analysis, neuroscience, representations, and memory were the motor themes that played an essential role within the structure of the field. fMRI and EEG as neuroimaging techniques appeared as emerging themes. These results are consistent with the findings from VOSviewer analysis during the first period. Wayfinding also appears as a relatively new emerging theme during this period.

##### Second Period

**Figure 7:**
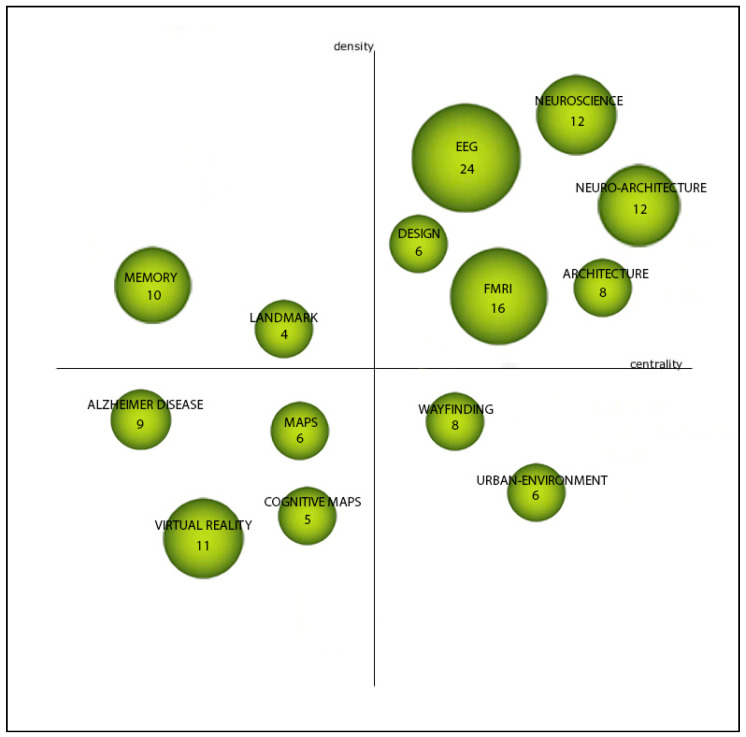
Major themes of the second period (strategic diagrams, see Section 2.3 and Figure 2 for description of the nodes and quadrants).

Figure 7 shows the SciMAT analysis for the strategic diagram during the second period (2016–2021). This period shows a remarkable increase in the number of annual publications in the field. Fourteen major themes emerged in this period. Overall, the thematic focus of the field was diversified. The themes neuroarchitecture, EEG, and fMRI stand out as the main themes and this demonstrates a major shift in the thematic focus toward the importance of brain imaging techniques and the emergence of neuroarchitecture as a discipline. The results show that the field begins to consolidate around the major theme of neuroarchitecture. It is also interesting that in this period most of the themes are either ‘motor’ or ‘emerging or declining’ themes. Design and architecture are other newly emerged motor themes that indicate there has been more attention paid to the interrelationships between neuroscience and architecture/design in this period. The large number of publications over the past six years supports these findings. It also can be seen that virtual reality and Alzheimer’s disease appear as newly emerging themes in this period.

### 3.3. Influential Journals

**Figure 8:**
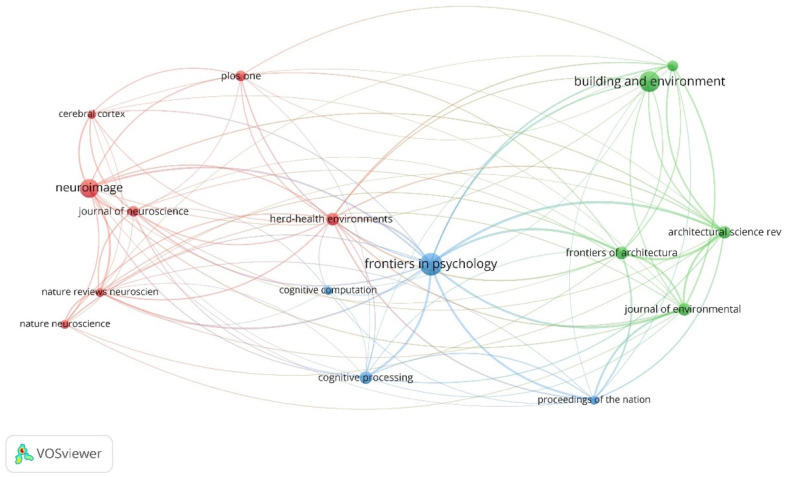
Co-citation analysis by cited sources.

Van Eck and Waltman [41] show that “co-citation is a link between two items that are both cited by the same document”. In this section, co-citation analysis has been used to identify the most influential journals in the field. The number of documents, citations and total link strength of the most prominent journals can be found in Table S1 of the Supplementary Materials. Figure 8 shows the co-citations analysis results by cited sources. The results indicate that three main journals in this field are the *Frontiers in Psychology*, *Building and Environment* and *Neuroimage*. This analysis also shows that there are three main clusters of journals which have a close relationship with the clusters identified in the co-occurrence analysis. The largest cluster (red color) revolves around neuroscience and biology, related to the red cluster of term co-occurrence analysis. The most prominent journals of this cluster are *Neuroimage*, *Herd Health Environments* and *Journal of Neuroscience*. Furthermore, the second-largest cluster (green color) mainly focuses on architecture and the built environment like the purple one in co-occurrence analysis. *Building and Environment*, *Architectural Science Review* and *Frontiers of Architectural Research* are three main journals of this cluster. The last but not least cluster (blue color) has mainly focused on the relationship between biology, cognition, and psychology associated with the green one in term co-occurrence analysis. The most prominent journals of this cluster include *Frontiers in Psychology*, *Cognitive Processing* and *Cognitive Computation*. This analysis also indicates that *Nature Reviews Neuroscience*, *Journal of Neuroscience* and *Nature Neuroscience* with 3935, 775, and 686 citations were the most cited journals, respectively.

### 3.4. Major Contributing Countries

**Figure 9:**
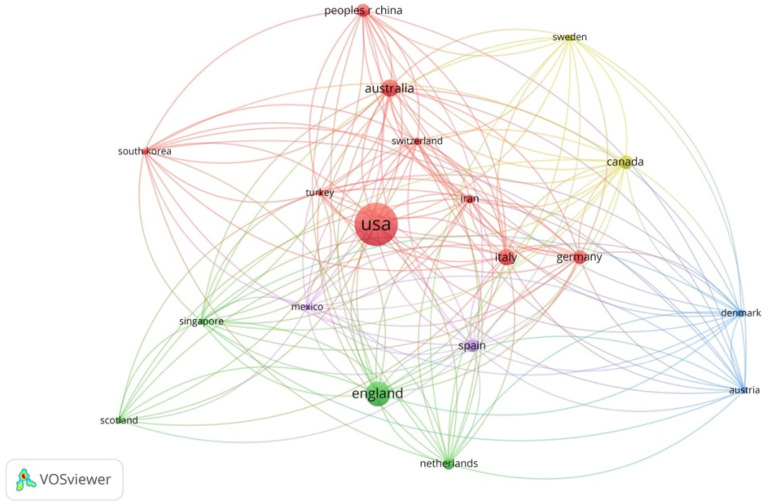
Co-citation analysis by countries.

A bibliographic coupling analysis was conducted to identify the most influential countries with the highest contribution in the field of neuroarchitecture. A bibliographic coupling link is a link between two items that both cite the same document [41]. According to the VoS database, miscellaneous authors from 19 countries have contributed to publishing 295 papers in the phase under review. Table S2 of the Supplementary Material presents the number of documents, number of citations, and total link strength in the most prominent countries in this field. Figure 9 shows the result of bibliographic analysis for the most prominent countries. The size of nodes indicates the number of publications in this area in that specific country. While developed countries have mostly contributed to research in the field, it can be seen that some research is being carried out in other countries, namely Iran, Turkey, and Mexico. The results of the analysis indicate that the United States is the most prominent country in this field by 103 publications and 31.5% of the total documents. England, Australia, Italy, Canada, and Germany by 42, 24, 22, 17, and 17 are other developed countries that have had a significant contribution to the field. Literature shows different cultural backgrounds impact neural activity in humans. Culture is stored in the human brain; it is uniquely evolved for gaining cultural capacities such as language and morality [78]. Cupchik et al. [79] also indicate that the level of literacy, emotional intelligence, and specific social and cultural attributes impact the esthetic perception of humans in neural studies. Therefore, because of cultural differences in different countries, there is a critical need for further studies in developing countries, especially Asian and African countries, to investigate the differences and provide a holistic overview of the research landscape.

### 3.5. Prominent Publications

**Figure 10:**
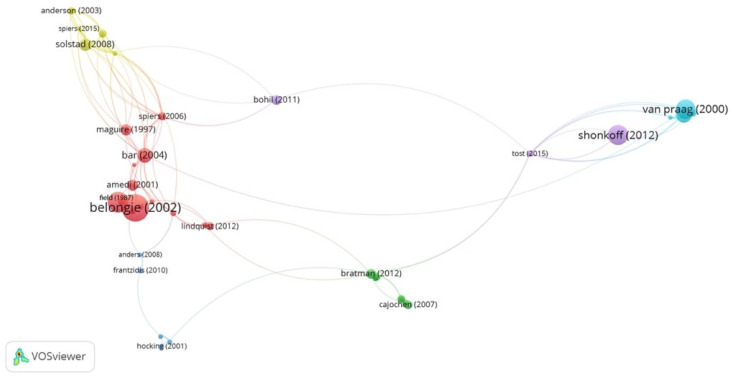
Co-citation analysis by cited references.

The most prominent publications in the field were also identified by co-citation analysis. Figure 10 demonstrates the analyses of cited references. The most cited references are listed in Table S3 of the Supplementary Materials. The analysis of most cited references indicates that there are two main clusters of most prominent publications (red and yellow). The red cluster shows studies revolving around object recognition, computational vision, and visual perception (e.g., shape matching and object recognition using shape [80], relations between the statistics of natural images and the response properties of cortical-cells [81], recalling routes around London: activation of the right hippocampus in taxi drivers [82]), and visual objects in context [83]) are associated with the blue cluster in Section 3.1 that is related to perception, recognition, and neuroimaging. The other main cluster (yellow) includes studies that have mainly focused on memory and spatial representation (e.g., representation of biometric borders in the entorhinal cortex [84], thoughts, behavior, and brain dynamics during navigation in the real world [85], and heterogeneous modulation of place cell firing by changes in context [86]). This cluster also shows a correlation with the yellow cluster in analysis in Section 3.1 that focuses on memory, spatial navigation, wayfinding, and hippocampus. In addition to these studies which belong to these main clusters, the lifelong effects of early childhood adversity and toxic stress [87], neural consequences of environmental enrichment [88] and enriched environments, experience-dependent plasticity, and disorders of the nervous system [89]) are also among the most influential studies in this field.

### 3.6. Prominent Authors

**Figure 11:**
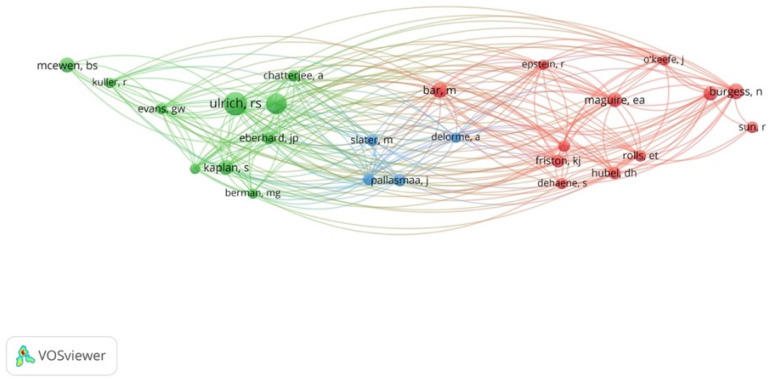
Co-citation analysis by authors.

Co-citation analysis based on cited authors was conducted to identify the most prominent authors in the field of neuroarchitecture. Table 3 shows the list of the most prominent authors in this field by a minimum of 20 citations. These influential authors and their relationships also have been shown in Figure 11. It can be clearly seen that there are three main clusters according to the author’s expertise: green, red, and blue. The authors’ work in the green cluster has mainly revolved around environmental behavior and the impact of the built environment on the cognition and emotion of humans (such as studies by Roger Ulrich, Stephan Kaplan, Marc G. Berman, Anjan Chatterjee). The red cluster indicates the authors that are experts in the field of cognitive maps, spatial perception, and navigation (e.g., Eleanor A. Maguire, Moshe Bar, Karl Friston, Neil Burgess). This cluster to a great extent is consistent with the red cluster of the previous section analysis as well as the blue cluster of Section 3.1.

## 4. Discussion

Despite a fast increase of the number of publications in the field of neuroarchitecture, still missing is a comprehensive review of the research landscape of the field and its evolution. There are some systematic reviews that have provided a framework for the existing knowledge of the field of neuroarchitecture; however, there has not been any study that investigates the evolution of the field and identifies the current trends. This study presents a bibliometric analysis of research in the field of neuroarchitecture to provide better insight on the evolution of knowledge, essential themes, and current trends. This paper evaluates global research trends in neuroarchitecture studies published within the three decades from 1992 to 2021. In this study, analysis captures temporal trends in research priority topics and provides foundational journals and papers, geographical representation, co-authorships linkages, and research collaboration clusters. While there is an increasing interest in the subject of how the human brain/mind responds to different environmental stimuli and also how this may benefit the field of architecture, engineering, and landscape design, very little is known at this time. The present paper provides an overview of the neuroarchitecture literature to identify major focus areas and key sources. Therefore, an in-depth discussion is presented in the following sections based on the aforementioned scientometric results of neuroarchitecture literature.

### 4.1. Theoretical Implications

Even though the topic of neuroarchitecture has drawn attention in the literature, there has been no holistic review of the current state of neuroarchitecture literature and its evolution that addresses emerging concepts in the field. A bibliometric analysis and visualization review has been carried out to provide the overall trends and hot spots in the field of neuroarchitecture. The main objective of this study was to provide a better understanding of the state of knowledge in the field through mapping the knowledge domain and highlighting emerging trends during different periods. As has been illustrated in the text, a vast body of literature of the built environment leading to collaborations between architecture and neuroscience has been published over the past three decades, especially after 2016. This paper, spanning from 1992 to 2021, presents the leading research authors, countries, and publications as well as dominant research themes and priorities based on a series of cooperation analyses. The knowledge of neuroarchitecture and analysis of evolution which contributes to the cross-disciplinary field of neuroscience and architecture provide the general required conceptual framework to find a common ground for future studies. The existing work provides a valuable and seminal reference for designers and architectures.

The findings of this literature mapping show that there have been only 291 publications, of which 48.62% were carried out in the preliminary period (1992–2015). There has been a fast growth in research in the field. Within the developing period (2016–2021) 51.38% of studies have been published. Accordingly, this shows that there is an average annual growth in publications in the field. The results also show that there are only six countries (USA, England, Australia, Italy, Canada, and Germany) that have majorly contributed to the research in the field. These countries together are accounted for 68.8% of all publications. These countries also host many significant scientists who have been working on this subject matter in recent years. While mentioned countries have made more contributions, it can be seen that a large body of research has been produced in developing countries; however, many countries, especially Asia and Africa, are lacking either in author cooperation or country cooperation to provide a scientific body of evidence on an international scale.

Despite the rapid growth in recent years, the development of the neuroarchitecture literature has been slow. When it comes to dividing the analysis into two sub-periods (1992–2015 and 2016–2021), the result shows that during the first period, the intellectual base was limited. However, in the second period, the intellectual base started to grow and consolidated around the major keywords mentioned in the preliminary period. The results showed that during the first period, studies mainly focused on the relationship between brain structure and function and human perceptual and cognitive process, while in second period literature has built a stronger bridge between architectural research and neuroscience. Major progress in the development of theoretical and methodological approaches has been achieved during this period and the paradigm shifted toward major keywords in neuroarchitecture such as EEG, fMRI, and virtual reality. Finally, over the past five years, the intellectual base has increasingly expanded and provides designers, educators, and psychologists more knowledge about indicators, tools, and methods to evaluate the relationship between architectural features and human experience. In particular, this research may provide useful insights into the trends in the field for designers and architects. The fast-growing neuroarchitecture field requires action to better understand the knowledge structure and trends. This requirement is well-recognized and there are now numerous publications and frameworks aiming to provide solutions on how to use neuroscience in architecture.

Findings from the science mapping method (strategic diagrams) using the SciMAT software tool were in line with the results of bibliometric analysis. The results of science mapping showed that the intellectual base of the field was relatively fragmented in the first period (1991–2015). However, the second period (2016–2021) showed a more diverse intellectual base as some of the themes were consolidated in this period. It should be noted that the diversity of the intellectual base is expected given the multi-faceted and dynamic nature of neuroarchitecture. The findings show that there was a paradigm shift toward the interrelationship between architecture and neuroscience. EEG and fMRI emerged as the motor themes in the second period. The results also show that virtual reality and Alzheimer’s disease appeared as newly emerging themes in this period.

### 4.2. Practical Implications

The results of this study can shed light on the evolution of the neuroarchitecture literature. The results of this study can be useful for researchers from multidisciplinary fields, graduate students, and scholars that are not sufficiently familiar with the research landscape and evolution of the field. Findings from this review can provide researchers with better insights to detect the main themes and understand the evolution of the research focus over time. The findings of this study also inform educators, researchers, and practitioners in the field regarding the paradigm shift towards the interrelationships between neuroscience and architecture, EEG and fMRI as motor themes, as well as emerging themes such as virtual reality and Alzheimer’s disease.

### 4.3. Limitations

While this study sheds light on the neuroarchitecture literature to identify major focus areas and key sources, some limitations should be noted. First, while VOSviewer appears to be an effective tool for literature mapping and visualization, this should not imply that science mapping and bibliometric analysis can replace systematic and integrative reviews. Second, since the VOSviewer tool in this study utilized keywords, publications titles, and abstracts for the screening of the articles, in future the use of more advanced techniques in machine learning and literature mapping/mining are essential to be able to review the full text of the publications. This might result in in-depth analysis when it is combined with integrative and systematic reviews. Third, interpretation of the identification of thematic clusters and term co-occurrences must be conducted with caution. In such analysis, the co-occurrences’ frequency and the links between the topics might not sufficiently explain and specify the nature of the relationships between the topics and keywords. Despite these limitations, the identification of the themes and trends resulting from the bibliometric mapping analysis can guide readers to more in-depth reviews. Finally, currently bibliometric analysis is only able to analyze the peer-reviewed journal publications indexed in scientific databases such as WoS and Scopus. It should be noted that while Scopus might cover a wider and inclusive content in some areas, WoS provides a deeper search and analysis of citation by source. The main reason that WoS was used for this study was that it is more suitable for large-scale citation analyses. WoS provides access to a large volume of scientific literature published in the past (from 1900), whereas in Scopus, citation coverage only extended to 1970 [90].

Future studies may include gray literature such as books, book chapters, reports, and conference proceedings papers, and other publications. It is also should be noted that while this study used VOSviewer to present informative tables, it is possible to manually make informative tables, with calculated h-indexes for authors, institutions, and cited resources. Overall bibliometric analysis studies do not replace systematic reviews in the field of study; instead, they complement each other. Bibliometric analysis and science mapping are different from systematic and integrative reviews as they aim to show the mapping of knowledge, overall landscape, and evolution of the topics over time.

## 5. Conclusions

This study provides insights to advance our understanding of the published literature related to neuroarchitecture and identify the topics of interest and evolution of the field over time. Future studies are recommended to increase our comprehension of specific issues. Future studies might also investigate how core concepts and trends can assist in the identification of the relationship between architecture and neuroscience and develop guidelines to design better environments that positively impact health and well-being.

## Figures and Tables

**Table 1:** Term co-occurrence analysis (top 20 most frequently occurring terms 1992–2021).

Keyword		Occurrences	Percentage	Total Link Strength
1	Architecture	43	5.2	181
2	Neuroscience	41	5	154
3	Brain	36	4.3	139
4	Brain responses	30	3.6	120
5	EEG	30	3.6	118
6	Memory	24	3	80
7	Design	23	2.8	101
8	Perception	23	2.8	92
9	fMRI	23	2.8	78
10	Stress	20	2.4	62
11	Representations	19	2.3	81
12	Cognition	19	2.3	75
13	Virtual reality	18	2.2	92
14	Environments	18	2.2	77
15	Hippocampus	18	2.2	61
16	Attention	17	2	74
17	Information	17	2	66
18	Performance	17	2	62
19	Experience	16	1.9	86
20	Wayfinding	15	1.8	55

**Table 2:** The most frequently used keywords for early research (1992–2015) and developing research (2016–2021).

	Keyword Preliminary Period (1992–2015)	*n*	%		Keyword Developing Period (2016–2021)	*n*	%
1	Memory	15	5.4	1	Neuroscience	31	7
2	Brain	13	4.7	2	Architecture	31	7
3	Architecture	12	4.4	3	EEG	24	5.4
4	Representations	12	4.4	4	Brain	23	5.2
5	Hippocampus	12	4.4	5	Brain Response	19	4.3
6	Brain responses	11	4	6	Design	17	3.8
7	Neuroscience	11	4	7	fMRI	16	3.6
8	Information	10	3.6	8	Virtual Reality	15	3.4
9	Prefrontal cortex	9	3.2	9	Perception	15	3.4
10	Cognition	9	3.2	10	Performance	14	3.2
11	Model	9	3.2	11	Experience	13	2.9
12	Stress	9	3.2	12	Attention	13	2.9
13	Perception	8	2.9	13	Environments	12	2.7
14	Neural	7	2.5	14	Stress	11	2.5
15	fMRI	7	2.5	15	Built environment	10	2.3
16	Cortex	7	2.5	16	Esthetics	10	2.3
17	Emotion	6	2.2	17	Wayfinding	10	2.3
18	Orientation	6	2.2	18	Cognition	10	2.3
19	Place cells	6	2.2	19	Memory	9	2
20	EEG	6	2.2	20	Emotion	8	1.8
21	Recognition	6	2.2	21	Color	7	1.6
22	Design	6	2.2	22	Behavior	7	1.6
23	Health	6	2.2	23	Neuro architecture	7	1.6
24	Environments	6	2.2	24	Light	7	1.6
25	Receptive fields	5	1.8	25	Cognitive architecture	7	1.6
26	Path integration	5	1.8	26	Model	7	1.6
27	Wayfinding	5	1.8	27	Recognition	7	1.6
28	Neurons	5	1.8	28	Representations	7	1.6
29	Spatial navigation	4	1.4	29	Information	7	1.6
30	Networks	4	1.4	30	Environmental psychology	6	1.3
31	Object recognition	4	1.4	31	Exposure	6	1.3
32	parietal cortex	4	1.4	32	Benefits	6	1.3
33	Esthetics	4	1.4	33	Organization	6	1.3
34	Attention	4	1.4	34	Hippocampus	6	1.3
35	Dynamics	4	1.4	35	Health	6	1.3
36	Embodiment	4	1.4	36	Preference	5	1.1
37	Organization	4	1.4	37	Judgments	5	1.1
38	Inferotemporal cortex	4	1.4	38	System	5	1.1
39	Features	4	1.4	39	Cortex	5	1.1
40	light	4	1.4	40	Impact	5	1.1
41	Pain	4	1.4	41	Prefrontal cortex	5	1.1

**Table 3:** Highly cited authors.

Author	Citations	Total Link Strength
Ulrich, R.S.	72	424
Vartanian, O.	60	412
Burgess, N.	39	200
Bar, M.	38	200
Mcewen, B.S.	37	20
Kaplan, S.	36	280
Okeefe, J.	35	177
Maguire, E.A.	33	156
Friston, K.J.	30	71
Eberhard, J.P.	27	139
Hubel, D.H.	27	49
Pallasmaa, J.	27	121
Mallgrave, H.F.	26	138
Sun, R.	26	30
Chatterjee, A.	25	286
Slater, M.	25	134
Gibson, J.J.	24	99
Rolls, E.T.	24	85
Evans, G.W.	23	196
Dehaene, S.	22	50

## Data Availability

Not applicable.

## Supplementary Materials

The following are available online at https://www.mdpi.com/article/10.3390/ejihpe11040099/s1, Table S1: Co-citation analysis by cited sources, Table S2: Countries with the largest number of publications. Table S3: Ten most cited references.
