# Supplementary material for: Neuroarchitecture Assessment: An Overview and Bibliometric Analysis
Source: Eur J Investig Health Psychol Educ. 2021 Nov 5;11(4):1362–87. doi: 10.3390/ejihpe11040099 (PMC8628715; doi:10.3390/ejihpe11040099)
Supplement: Supplementary file 1 [file ejihpe-11-00099-s001.zip › ejihpe-1381433-supplementary.pdf]

Supplementary material for the article titled:

Neuroarchitecture Assessment: An Overview and Bibliometric Analysis

Items in this Supplementary document:

- The search strings
- Table S1: Co-citation analysis by cited sources
- Table S2: Countries with the largest number of publications.
- Table S3: Ten most cited references

The search string:

TS= (("architecture" OR "built environment" OR "architectural space" OR "interior space" OR "environment Design" OR "physical environment") AND ("neuroscience" OR "neuro architecture" OR "neuroimaging" OR "brain imaging" OR "Brain Reponses" OR "Neural Activation" OR "neural responses"))

Table S1. Co-citation analysis by cited sources (top 20 most influential journals)

Source	Documents	Citations	Total link strength
Journal of Frontiers in Psychology	11	165	337
Building and Environment	10	383	61
Neuroimage	9	591	119
Architectural Science Review	6	49	201
Cognitive Processing	6	51	75
Frontiers of Architectural Research	6	16	173
Health Environments Research Design (HERD)	6	49	116
Journal of Environmental Psychology	6	266	171
Design	5	0	0
International Journal of Environmental Research and Public Health	5	51	95
Journal of Neuroscience	5	775	66
Plos One	5	20	32
Cerebral Cortex	4	395	38
Cognitive Computation	4	27	12
Nature Neuroscience	4	686	31
Nature Reviews Neuroscience	4	3935	88

Table S2. Countries with the largest number of publications.

Country	Documents	Citations	Total link strength
Usa	103	9849	8940
England	42	2046	5157
Australia	24	1445	3171
Canada	17	570	4265
Italy	22	554	1632
Germany	17	471	2203
Austria	5	431	1894
South Korea	5	394	281
Switzerland	7	340	394
Spain	16	330	3378
Denmark	7	277	2199
Scotland	5	253	584
China	15	232	1027
Netherlands	12	183	949
Sweden	6	126	108
Singapore	5	76	643
Iran	9	28	1622
Turkey	5	20	559
Mexico	5	18	438

Table S3. Ten most influential publications

Title	Co-citations	total link strength	Reference
Shape matching and object recognition using shape contexts	3680	1	[79]
Relations between the statistics of natural images and the response properties of cortical-cells	1981	1	[80]
The Lifelong Effects of Early Childhood Adversity and Toxic Stress	1822	9	[86]
Neural consequences of environmental enrichment	1647	32	[87]
Enriched environments, experience-dependent plasticity and disorders of the nervous system	1042	47	[88]
Visual objects in context	891	25	[82]
Representation of Geometric Borders in the Entorhinal Cortex	559	21	[83]
Recalling routes around London: activation of the right hippocampus in taxi drivers	449	9	[81]
Visuo-haptic object-related activation in the ventral visual pathway	441	9	[90]
The impacts of nature experience on human cognitive function and mental health	369	14	[91]
